# Beneficial Pharmacokinetic Drug Interactions: A Tool to Improve the Bioavailability of Poorly Permeable Drugs

**DOI:** 10.3390/pharmaceutics10030106

**Published:** 2018-07-26

**Authors:** Werner Gerber, Johan D. Steyn, Awie F. Kotzé, Josias H. Hamman

**Affiliations:** Centre of Excellence for Pharmaceutical Sciences, North-West University, 2520 Potchefstroom, South Africa; wgerber99@gmail.com (W.G.); dewald.steyn@nwu.ac.za (J.D.S.); awie.kotze@nwu.ac.za (A.F.K.)

**Keywords:** bioavailability, efflux inhibition, pharmacokinetic interactions, tight junction modulation, enzyme inhibition

## Abstract

Simultaneous oral intake of herbs, supplements, foods and drugs with other drug(s) may result in pharmacokinetic or pharmacodynamic interactions with the latter. Although these interactions are often associated with unwanted effects such as adverse events or inefficacy, they can also produce effects that are potentially beneficial to the patient. Beneficial pharmacokinetic interactions include the improvement of the bioavailability of a drug (i.e., by enhancing absorption and/or inhibiting metabolism) or prolongation of a drug’s plasma level within its therapeutic window (i.e., by decreasing excretion), whereas beneficial pharmacodynamic interactions include additive or synergistic effects. Mechanisms by which pharmacokinetic interactions can cause beneficial effects include enhancement of membrane permeation (e.g., structural changes in the epithelial cell membranes or opening of tight junctions), modulation of carrier proteins (e.g., inhibition of efflux transporters and stimulation of uptake transporters) and inhibition of metabolic enzymes. In the current review, selected pharmacokinetic interactions between drugs and various compounds from different sources including food, herb, dietary supplements and selected drugs are discussed. These interactions may be exploited in the future to the benefit of the patient, for example, by delivering drugs that are poorly bioavailable in therapeutic levels via alternative routes of administration than parenteral injection.

## 1. Introduction

Many patients are taking food, herbs, supplements and/or over-the-counter health products together with their prescribed medications [1,2]. Concomitant use of these products may lead to food-drug, herb-drug, supplement-drug and/or drug-drug interactions [3,4]. Although these interactions are usually complicated and unpredictable, it is known that they can indeed influence the pharmacodynamics and/or pharmacokinetics of some co-administered drugs [1,2,3,4,5]. Pharmacodynamic interactions mainly involve diverse reactions at receptor sites resulting in antagonistic or synergistic effects as well as causing changes in physiological environments, while pharmacokinetic interactions affect the absorption, distribution, metabolism and excretion of the co-administered agent (e.g., food component, herb, supplement, health product ingredient) and/or co-administered drug [1,2,4,5]. These interactions have been associated with negative effects, which include either adverse effects/toxicity or insufficient plasma concentrations of the co-administered drug(s). However, it is possible that these interactions can improve the pharmacodynamics and/or pharmacokinetics of a co-administered drug [1,3,4]. 

**Figure 1:**
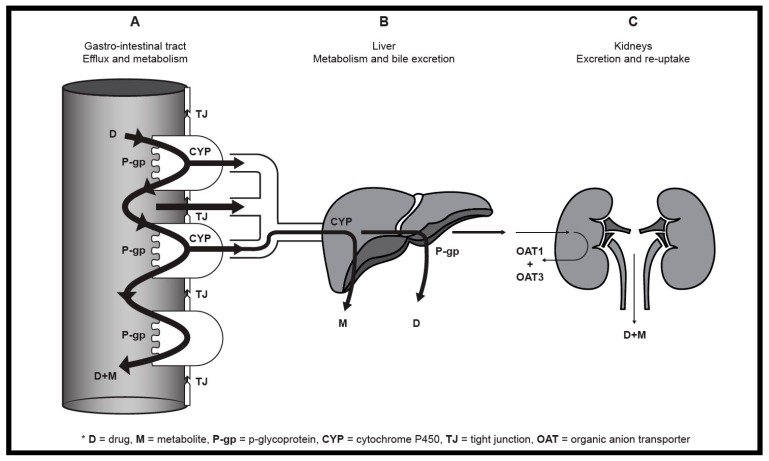
Schematic illustration of the major sites where pharmacokinetic drug interactions can occur after oral administration including the (**A**) gastro-intestinal tract, (**B**) liver and (**C**) kidneys.

Figure 1 depicts a schematic illustration of the major areas where pharmacokinetic interactions may occur after oral drug administration. 

The most prominent reasons why drugs present with poor oral bioavailability include low solubility, extensive pre-systemic metabolism, poor membrane permeability and active efflux transportation (Figure 1A,B). All these characteristics cause a reduction in the total amount of drug that will become available in the plasma [2,4,6]. Deliberate inhibition of pre-systemic metabolism or efflux can achieve improved bioavailability and prolonged half-lives of poorly bioavailable drugs with consequently less frequent dosing and a lower total required dose (i.e., inhibition of P-glycoprotein [P-gp], cytochrome P450 [CYP450] and organic anion transporters [OAT] 1 and 3) [1,4,6]. This will result in cost savings and improved patient compliance [1]. Figure 1 indirectly also illustrates different possible areas that may be targeted for beneficial pharmacokinetic interactions that can lead to increased drug bioavailability. The mechanisms of these interactions as well as examples of such interactions are discussed in more detail in the sections below.

## 2. Mechanisms of Pharmacokinetic Drug Interactions

### 2.1. Carrier-Mediated Transporters

Carrier-mediated transporters refer to a collection of proteins that are found on cellular membranes throughout the body which are mainly responsible for processes that mediate cellular uptake and transcellular permeation. These processes may either be via active transport or passive facilitated transport. Active transport refers to adenosine tri-phosphate (ATP)-dependent processes where a permeant is transported against its own concentration gradient, whereas passive facilitated transport occurs in the absence of ATP and with the concentration gradient of the permeant. Both types of transporters mainly allow the passage of solutes into cells and organelles, but due to the fact that each transporter can only carry one molecule at a time, the processes are saturable [7,8,9].

Active transport can occur via either primary-active or secondary-active transporters. Primary-active transporters hydrolyze ATP directly in order to be able to transport the permeant across a membrane. Members of the ATP-binding cassette (ABC) superfamily are examples of primary-active transporters. Secondary-active transporters are mainly ion pumps. Examples include the superfamily of transporters encoded by the solute-carrier (SLC) gene such as organic anion transporters (OATs). The indirect dependence on ATP is why they are referred to as secondary-active transporters [7,8,9]. 

The ABC superfamily of transporters is responsible for a broad range of ATP-dependent efflux transport of compounds with diverse chemical properties. The most abundantly distributed ABC transporter is P-glycoprotein (P-gp), also known as multidrug resistance protein 1 (MDR1), encoded by the *ABCB1* gene, which is found in the liver, kidneys, testes, on the intestinal brush border membrane and the blood-brain barrier [10,11,12]. Other ABC transporters include cystic fibrosis transmembrane conductance regulator (CFTR), transporter associated with antigen processing (TAP), breast cancer resistance protein (BCRP) and multidrug resistance-associated protein 1 (MRP1) of which the latter two are often found in cancerous tissues [7,10,11]. The principle function of these transporters is to efflux any xenobiotic compounds out of the cell, thus keeping the host safe of a wide range of toxic compounds. Many drug molecules also present with a high affinity to one or more of these transporters, thus leading to reduced bioavailability and/or pharmacological response (e.g., anti-cancer drugs are pumped out of tumor cells). The reason for decreased bioavailability after oral administration due to efflux is because the drug molecules are actively transported back towards the apical side of the intestinal epithelial cell membranes and into the lumen of the gastrointestinal tract [10,11]. By inhibiting ABC efflux transporters, it is therefore possible to increase the bioavailability of efflux transporter substrates [11,12].

One family of the SLC superfamily with clinical relevance is the OAT uptake transporters expressed mainly in the renal proximal tubules encoded specifically by four of the *SLCO* gene families [7,13,14,15]. OAT1 and OAT3 are situated on the basolateral side of the membrane and are responsible for excretion of compounds by tubular secretion of metabolites and/or other xenobiotics from the blood to the urine (Figure 1C). In contrast, OAT4 is situated on the apical side of the membrane where it is responsible for re-absorbing anionic compounds from the urine to the blood [13,15]. Since certain drugs are mainly excreted via the kidneys, the OATs play a role in the reduction of plasma levels of such drugs via excretion. 

### 2.2. Metabolism

Pre-systemic metabolism is one of the first hurdles any orally ingested compound faces as it occurs in the gastro-intestinal tract and the liver (Figure 1A,B) before the compound reaches the systemic circulation. Inhibition of pre-systemic metabolic enzymes may result in more drug molecules reaching the site of absorption unchanged and unaltered leading to increased bioavailability [6]. Metabolism in the liver occurs in two main phases; phase 1 is largely dictated by oxidation reactions, whereas phase 2 consists of conjugation processes [16,17]. CYP450 is the most abundant metabolic enzyme system in the human body and this superfamily of enzymes are responsible for over 60% of all drugs’ metabolism by means of phase 1 reactions (i.e., oxidation, reduction and hydrolysis) [1,4,16,18]. The quantity of sub-families of CYP450 differs in different tissues. Most notably, CYP3A4/5 consists of approximately 82% of CYP450 in the small intestines, but represents only 39% of CYP450 in the liver. On the other hand, where CYP2C9 is only represented by 13% in the small intestine, 25% of this enzyme is found in the liver. Furthermore, CYP2B6, CYP2D6, CYP2A6, CYP2E1 and CYP1A2 make up 1%, 2%, 6%, 9% and 18% of enzymes present in the liver, respectively [4]. Other enzyme families active during phase 1 metabolism include flavin-containing monooxygenases and epoxide hydrolases [17]. CYP450 is responsible for decreasing the bioavailability of drugs, except in the limited cases of pro-drugs where metabolites are the pharmacologically active compounds. Many compounds have been identified to be the inhibitors of CYP450, thus actively increasing the bioavailability of drugs that are substrates of CYP450 enzymes. CYP450 inhibition may therefore have the risk of increasing the toxicity of certain drugs, but have the potential to be deployed to benefit poorly bioavailable drugs that are susceptible to extensive pre-systemic metabolism [1,4,16,18]. 

Only after phase 1 metabolism introduced functional groups to molecules being metabolised, can the conjugating processes of phase 2 begin, by which water solubility of the conjugate is greatly increased. Phase 2 metabolism may occur through glucuronidation mediated by uridine 5′-diphospho-glucuronosyltransferase (UGT), sulfation mediated by sulfotransferases, methylation mediated by methyltransferases, N-acetylation mediated by N-acetyltransferases and glutathione conjugation by gluthathione-S-transferases [17]. One of the most studied phase 2 metabolic enzymes remains UGT, which is a superfamily of enzyme that plays a major role in the conjugation of drugs in the liver by means of glucuronidation. By mediating such glucuronidation processes, UGT also detoxifies endogenous toxins like bilirubin, bile acids and fat-soluble vitamins [19,20,21,22]. It has been previously reported that many drugs are substrates for UGT metabolism, but also that numerous phytochemicals are glucuronidated in the body by UGT [23]. The possibility for drug interactions exist by means of competitive inhibition of UGT, which may lead to increased bioavailability of drugs that are substrates for this enzyme.

## 3. Potential Beneficial Pharmacokinetic Interactions between Drugs and Other Substances

As mentioned before, pharmacokinetic drug interactions involving modulation of certain active transporters and metabolic pathways by co-administered substances may lead to unwanted outcomes. When efflux transporters and metabolic enzymes are induced, it may lead to achievement of sub-therapeutic drug plasma levels that are associated with pharmacological ineffectiveness. On the other hand, when efflux transporters and metabolic enzymes are inhibited, it may lead to improved bioavailability and more effective therapeutic outcomes if the drug plasma level is within the therapeutic window. However, achievement of supra-therapeutic drug plasma levels are associated with adverse effects and possibly also toxicity [24,25]. Although the outcomes of arbitrary drug interactions are often unpredictable, unwanted and complicated, which in some cases may be serious and have life-threatening consequences [4], it may be used in a controlled manner to the therapeutic benefit of patients. It is therefore not surprising to note that herb-herb and herb-drug combinations are prescribed for this reason [1,3,18].

The focus in this paper is on beneficial pharmacokinetic interactions and specifically on more recent discoveries in this field, whilst well-known interactions such as those caused by grapefruit, green tea, probenecid and St. John’s wort are also mentioned. The discussions in this paper are based on recently reported (most studies within 5 years) scientific studies that were obtained from an electronic literature search using ScienceDirect, GoogleScholar and Scopus as search engine. The studies were selected to represent additive agents from different broad categories including food, herbs, dietary supplements and drugs that were investigated for pharmacokinetic interactions with a drug/marker molecule in different models including ex vivo, in vitro and/or in vivo. Only studies that reported on potential beneficial pharmacokinetic interactions (i.e., improved drug bioavailability or increased membrane permeation) were selected and are summarised after discussion in Table 2 (Section 4).

### 3.1. Food-Drug Interactions

The term “food” can be defined as materials that consist of different chemical components such as proteins, carbohydrates and fat that can be used in an organism as nutrients to sustain growth, repair and maintain vital processes as well as furnish energy [26]. It is also known that the peroral route of drug delivery is by far the most used and preferred route of drug administration due to its safety, ease of administration and fixed dosage administration. Orally ingested drugs may come into contact with ingested food. Food can affect drug absorption via different mechanisms such as changing gastric emptying time, the pH and residence time of the drug in the gastrointestinal tract, the hepatic blood flow and by modulating efflux transporters as well as pre-systemic metabolism [4]. One of the classical pharmacokinetic interactions of food components with drugs is that of grapefruit juice with nifedipine [27]. Certain recently discovered pharmacokinetic interactions of selected food components that may enhance drug bioavailability to the benefit of the patient are discussed in the sections below.

#### 3.1.1. Black Pepper

Piperine (1-piperoylpiperidine) is a major bioactive alkaloid constituent of long pepper (*Piper longum* L.) and black pepper (*Piper nigrum* L.) [28,29,30]. Piperine is believed to have anti-inflammatory, anti-hypertensive and hepatic protective properties [29,30]. It is often combined with other herbal medicines such as curcumin and was found to enhance the absorption of these co-administered herbs and drugs [31].

Mechanistic studies indicated that piperine is able to inhibit the active efflux protein transporter, P-gp, as well as the CYP3A4 enzyme [28,29]. These studies were conducted using in vitro models, such as the human colon carcinoma cell line (Caco-2) [28], and it was only relatively recently evaluated by using in vivo animal models [29]. Peroral administration in a male Sprague-Dawley rat model was mainly used due to the obvious ingestion route of pepper but also due to the high concentrations of P-gp transporters and CYP3A4 enzymes present in the enterocytes, thus presenting a large area for possible interactions [29]. Piperine was also investigated as a means to increase the bioavailability of another bioactive herbal compound, emodin. Piperine increased the area under the plasma concentration time curve (AUC), which indicates an increase in the total amount of drug absorbed, and maximum plasma concentration (*C*_max_) of emodin by 221% and 258%, respectively. Simultaneously, the AUC and *C*_max_ of emodin glucuronide, a metabolite of emodin, were reduced by 267% and 369%, respectively. These results indicate that piperine is able to inhibit glucuronidation of emodin significantly [29].

Another example is that of docetaxel, which is an anti-cancer drug, but has limited clinical application due to its low bioavailability. Li et al. [30] conducted a study to determine the pharmaco-kinetic profile of docetaxel in the presence of piperine. Combined use increased the initial concentration (C_0_) of docetaxel three times and the AUC with 61.7%. Simultaneously, piperine’s t_1/2_ increased significantly while a moderate increase was observed in its C_max_ and AUC_0-t_ [30].

It is helpful to know that concentrations of piperine needed for significant pharmacokinetic interactions are non-toxic to intestinal epithelial tissues as indicated by TEER and [^3^H]inulin transport studies [28,29]. With this knowledge, Shao et al. [31] developed a self-emulsifying drug delivery system (SEDDS) to enhance bioavailability and solubility of piperine in order to develope a potentially useful system to clinically use piperine. In the formulation, the SEDDS increased the relative bioavailability of piperine by 625.7% compared to self-prepared capsules. It was also found that the formulation’s *C*_max_ and AUC_0–60h_ values were approximately five times greater than that of the capsules.

#### 3.1.2. Orange Juice

Some of the most abundant bioflavonoids in orange juice include hesperidin, nobiletin and tangeretin [32]. Furthermore, it has been reported to exhibit biological effects like anti-inflammatory, cholesterol lowering and neuroprotective activities [33]. Recently, studies showed that tangeretin and nobiletin increased intracellular concentrations of anti-cancer drugs in vitro due to inhibition of P-gp and BCRP [32,33]. Fleisher et al. [32] found that tangeretin and nobiletin significantly inhibited P-gp-mediated by increasing intracellular concentration of a small molecule tyrosine kinase inhibitor (TKI), dasatinib, by 314% and 476%, respectively when compared to the control of dasatinib alone [32]. Furthermore, the same study also reported a significant inhibition of BCRP-mediated transport. Intracellular concentration of dasatinib was increased by 845% and 949% for tangeretin and nobiletin, respectively when compared to the control of dasatinib alone [32]. The latter results are especially important because they are similar to results found with a potent known BCRP inhibitor, elacridar (5 µM), which increased intracellular concentrations of dasatinib by 782% [32].

Most TKIs are known to be substrates for both P-gp and BCRP and are also mainly delivered orally in a once daily dose. Therefore, the results show that potential beneficial interactions may arise by taking the TKIs with a specific diet. Orange juice contains an average concentration of 2.633 µM tangeretin and 6.345 µM nobiletin, therefore intestinal concentrations higher than their individual IC_50_ values for BCRP inhibition may be easily obtained [32]. 

Along with its cytotoxic effects, the effect that tangeretin had on P-gp was again confirmed in 2016 by efflux inhibition of rhodamine 123 (R123) and doxorubicin (DOX) across Caco-2 cells [33]. The study found that the efflux ratio values of both R123 and DOX were decreased to ≈1 in the presence of tangeretin, which is much lower than the control of ≈7 for both markers. Similar efflux inhibition of a known P-gp inhibitor, quinidine (20 µM), was achieved by tangeretin at a concentration of 2.51 µM. Accumulation of doxorubicin and flutax-2 in Caco-2 cells were also clearly portrayed in the presence of tangeretin by means of fluorescence microscopy [33]. This study also found that tangeretin is able to stimulate P-gp ATPase, while simultaneously inhibiting verapamil-stimulated ATPase activity. The authors postulated that tangeretin might competitively inhibit substrate binding sites on P-gp, which would explain the increase in ATPase activity with simultaneous decrease in transporting activity.

#### 3.1.3. Resveratrol

Resveratrol is a polyphenolic phytoalexin found abundantly in berries, grape skins and red wine. It has proven anti-oxidant and anti-inflammatory effects, thus leading to a wide range of health benefits, which include cardiovascular protection [5,34,35]. Pharmacokinetic studies in human volunteers indicated that resveratrol is able to inhibit CYP2C9 and CYP2E1 in vivo [34,35]. For CYP2C9 inhibition studies, diclofenac was used as a substrate and taken orally after 10 days of daily oral resveratrol treatment. Pharmacokinetic parameters of *C*_max_, AUC and half-life (t_1/2_) was determined after a 10-day treatment and these parameters were each found to be 68.2%, 98.8% and 57.1% higher than in the control group whom received diclofenac alone [35]. These results showed significant inhibition of CYP2C9 metabolism, which indicated that lower doses of diclofenac were needed for pharmacological effects and may therefore reduce the common known side-effects of diclofenac. Furthermore, chlorzoxazone is often used as a probe for specific CYP2E1 metabolism studies. Resveratrol was found to not only increase certain pharmacokinetic parameters of chlorzoxazone after 10 days of oral pretreatment, but also decreased the plasma concentration of a known metabolite, 6-hydroxychlorzoxazone. The *C*_max_, AUC and t_1/2_ of chlorzoxazone were increased by 54.2%, 72.2% and 34.8%, respectively [34]. In both scenarios, the clearance parameters, K_Cl_ and CL/F, were also decreased [34,35].

Furthermore, results exist that indicate that resveratrol can also inhibit various transporter proteins [5]. A comprehensive study by Jia et al. [5] showed that resveratrol inhibited P-gp, MRP-2 and organic anion transporter (OAT) 1 and 3. Methotrexate, a known substrate of P-gp, presented with increased intestinal absorption in vivo and in vitro in the presence of resveratrol. The AUC and *C*_max_ parameters were increased by factor 1.1 and 1.5, respectively, while simultaneously shortening the *T*_max_. Here it is accepted that intestinal P-gp- and MRP2-mediated transport was inhibited by resveratrol seeing that permeability studies using the rat everted sac method yielded similar results. However, after intravenous administration of methrotrexate along with resveratrol in rats, the t_1/2_ of methotrexate increased by 66% while clearance was decreased by 45%. The study further found that the urinary secretion of methotrexate statistically significantly decreased. Taking the aforementioned results into account, it is a good indication of the ability of resveratrol to inhibit OAT1 and OAT3 [5].

### 3.2. Herb-Drug Interactions

In the context of the current discussion, the term “herb” refers to a plant or plant part that is valued specifically for its medicinal, savory or aromatic qualities [36]. Herbal medicines are often used by patients in conjunction with their conventional medicines. This may happen without the prescribing physician knowing about it. Concurrent use of different remedies poses a potential risk for patients, especially with herb-drug interactions being capable of modulating pharmacodynamic and pharmacokinetic profiles of drugs [2,37,38]. Well known herb-drug interactions have been thoroughly described in the literature and includes interactions such as St John’s wort’s induction of P-gp expression and metabolism [2,30], UGT inhibition by milk thistle, saw palmetto and cranberry [23] and *Gingko biloba*’s pharmacodynamic brain protective properties [15]. Recently explored possible beneficial pharmacokinetic interactions regarding commonly used herbs are described in the following sections.

#### 3.2.1. Aloe Leaf Materials

*Aloe* is a genus of perennial succulent plants consisting of more than 400 species. As a medicinally versatile plant, aloe latex and gel have for centuries been used for its laxative and wound healing properties. Other medicinal activities include anti-cancer, anti-oxidant, anti-inflammatory, anti-diabetic, anti-ulcer and immunomodulatory effects. Materials from different aloe species are being investigated for new and innovative medicinal properties and applications [39,40].

The in vitro drug absorption enhancing effects of *A. vera* gel and whole leaf materials were first shown across intestinal epithelial cell (Caco-2) monolayers [41], which was attributed to opening of tight junctions between adjacent epithelial cells as indicated by a reduction in trans-epithelial electrical resistance. Other publications also reported the ability of aloe materials to increase intestinal membrane permeation of various drugs in vitro [42,43], which was also achieved when the aloe materials were incorporated into solid oral dosage forms (i.e., dual phase spherical bead system) [44]. It was found that *Aloe vera* liquid preparations could increase the bioavailability of vitamins in humans when co-administered [45]. Ojewole et al. [46] found that *A. vera* gel could also increase the buccal absorption of the anti-retroviral drug, didanosine, by as much as 11-fold [46]. They postulated that the mechanism responsible for the absorption enhancing effects may be because of increased residence time due to muco-adhesive abilities of aloe gel or that the polysaccharides in aloe are able to weaken the epithelial barrier by dismantling intercellular junctions. 

Three different species of aloe, namely *A. vera*, *A. ferox* and *A. marlothii* were deployed to study potential modulation of cimetidine efflux [37]. This in vitro study found that precipitated polysaccharides from *A. vera* gel inhibited the efflux of cimetidine (i.e., reduced the efflux ratio from 1.452 to 0.860), while the whole leaf and gel materials had no effect. Interestingly, *A. ferox* and *A. marlothii* showed the opposite effect with the gel and whole leaf materials inhibiting efflux, while the precipitated polysaccharides had seemingly induced efflux. The reason presented for this phenomenon was the different chemical composition of the three species [37]. 

*A. vera* and *A. marlothii* gel as well as *A. ferox* whole leaf materials were shown to improve ketoprofen diffusion across dermatomed skin [47]. Furthermore, different fractions of *A. vera* gel polysaccharides were found to inhibit indinavir metabolism in an in vitro LS180 cell model, while changing the pharmacokinetic parameters in vivo in male Sprague-Dawley rats after oral co-administration [38].

#### 3.2.2. *Salvia Miltiorrhiza*

*Salvia miltiorrhiza* is a traditional medicinal herb used for hundreds of years in China for an assortment of cardiovascular related diseases. Locally referred to as Danshen, the plant is commonly known as red sage in the Western world [48,49,50]. Due to its clinical effectiveness, there are currently more than 900 commercial preparations available containing Danshen in China [50]. Interactions of *S. miltiorrhiza* with other cardio-active drugs have been studied, which have shown that this herb may have a profound effect on drug displacement from serum albumin [48,49,50].

Chemical isolation and characterisation of *S. miltiorrhiza* have shown more than 60 phytochemical compounds to be present. Included in these compounds are water-soluble phenolic acids, salvianolic acid B and rosmarinic acid. These two phenolic acids are the most abundant and are believed to be the most bioactive in *S. miltiorrhiza* [49,50]. 

Serum albumin is the most abundant protein in the circulatory system. It acts as a carrier, which directly determines the pharmacokinetic and pharmacodynamic properties of the drug. Two binding sites, subdomain IIA (a.k.a. Sudlow I) and subdomain IIIA (a.k.a Sudlow II) are the two main areas of ligand binding [48,49]. Two studies conducted independently of one another have proven that salvianolic acid B can be bound to human serum albumin by interaction in the hydrophobic subdomain IIA cavity [48,49]. More recently, it was found that salvianolic acid B had such a high affinity for human serum albumin that it was able to displace site-specific warfarin from subdomain IIA, releasing more warfarin molecules into the plasma, which may lead to increased bleeding [50].

This is of potential importance as many drugs bind to serum albumin once absorbed into the blood stream. The efficacy of drugs also largely depends on the extent of albumin complexes as only the unbound fraction of the drug is available for pharmacological action or exposed for metabolism [48,49]. In the future, it may be possible to increase the pharmacological activity of highly bound drugs at lower doses by concomitant administration of *S. miltiorrhiza*.

#### 3.2.3. *Andrographis Paniculata*

Commonly referred to as green chiretta, *Andrographis paniculata* is an indigenous plant to South East Asia and has been deployed for centuries in traditional remedies to treat airway ailments, inflammation and more recently, acquired immunodeficiency syndrome (AIDS) [19,51]. In recent years, several different constituents of the plant have been identified and distinguished and consequently tested for any possible herb-drug interactions. A recent publication indicated the ability of an aqueous extract of *A. paniculata* to inhibit human liver glucuronidation activity [19]. This study found that metabolism of an UGT substrate, 4-methylumbelliferone, was significantly inhibited. Another extracted constituent of *A. paniculata*, named dehydroandrographolide (DAG), was found to inhibit P-gp mediated efflux of digoxin by as much as 70% in MDR1-MDCKII cells and 94% in Caco-2 cells [2]. The authors of the study postulated the mechanism for P-gp inhibition as competitive binding of DAG to transport sites, thus inhibiting the secretory transport of digoxin, while subsequently increasing the intracellular concentration of the drug. In the same study they also found that DAG significantly inhibited CYP3A4 [2].

Furthermore, arabinogalactan (an aqueous extract phytochemical component of *A. paniculata*) may also increase the plasma levels of co-administered drugs by displacing the drug molecules from serum albumin. It was found by fluorescent quenching that arabinogalactan bind to bovine serum albumin in a concentration dependent manner [51].

#### 3.2.4. Emodin

Emodin is an active constituent of multiple medicinal plants such as *Cassia angustifolia* (senna), *Aloe barbadensis* (aloe) and *Reum officinale* (rhubarb). It has been deployed as traditional medicine to assist with obesity-related problems, as an anti-diabetic agent and as a laxative. Recently it has been discovered that emodin may inhibit migration of cancer cells, but may not reach clinically significant plasma concentrations due to its low bioavailability as a result of extensive glucuronidation [29]. Emodin proved to be a strong inhibitor of P-gp mediated efflux by reducing in vitro digoxin efflux by 94% in MDR1-MDCKII cells and 82% in Caco-2 cells. This correlated well with the 51% increase in the AUC_total_ parameter of digoxin in the presence of emodin [2]. Due to emodin’s own possible anti-cancer effects it may hold great promise to administer emodin in conjunction with other anti-cancer therapeutics that are also substrates for efflux transporters.

### 3.3. Dietary Supplement-Drug Interactions

The term “dietary supplement” is specifically defined in this context as an orally ingested product that is intended to supplement a person’s diet and is not necessarily considered food (e.g., vitamins, minerals, herbs, amino acids) [52]. Understanding dietary supplement-drug interactions is especially important as a relatively large number (about 25%) of individuals that make use of dietary supplements on a regular basis, take it with one or more prescription drugs [20]. Furthermore, it is already known that dietary supplements may inhibit or induce enzymes resulting in altered bioavailability of drugs and subsequent adverse effects and/or loss of efficacy [18,20].

#### 3.3.1. Licorice (*Glycyrrhiza* Species)

*Glycyrrhiza*, a genus of plants commonly known as licorice, has been used in Asian countries since ancient times as a traditional medicine designated to treat allergies, virus infections and inflammation. Recently, glycyrrhizin was isolated from *Glycyrrhiza* plant species and has been deployed as sweetener and flavouring agent in prepared foods [15,16,18]. *Glycyrrhiza glabra*, *G. inflata* and *G. uralensis* are the three species of the plant that is mostly used in dietary supplements. Although the three species are often used interchangeably, they present with slightly different phytochemical constituents [18]. Studies identified six flavonoids extracted from *Glycyrrhiza* plants that presented with the capability of inhibiting CYP450 mediated metabolism. The extent of CYP450 inhibition by different phytochemicals from *Glycyrrhiza* species is shown in Table 1. 

Licochalcone A inhibited CYP450 in a time-dependent manner [16,18] and therefore the possibility exists that certain components of dietary supplements such as licorice may benefit the pharmacokinetic properties of extensively metabolized drugs. The variation in enzyme inhibition by different phytochemical components requires determination of the exact chemical compound in a given dietary supplement that causes the drug bioavailability enhancement effect.

A study conducted on the transporter inhibition potential of 18*β*-glycyrrhetinic acid, showed significant reduction (56%) of *p*-aminohippurate (PAH) transport via the hOAT1 transporter. This indicates the ability to decrease renal secretion and stimulate re-absorption into the blood [15]. The same study also showed a significant stimulation of estrone sulfate (ES) uptake via hOAT4 transporters. As the hOAT4 transporter is mainly responsible for re-uptake into the blood from the urine, stimulation thereof may be used as a beneficial interaction to maintain therapeutic levels of renally excreted drugs.

#### 3.3.2. Carotenoids

Carotenoids are yellow pigments found in many plant and animal species. A sub-group of these pigments, named xantophylls, are widely marketed as dietary supplements to be used for cancer prevention as well as in cardiovascular and chronic inflammatory diseases due to their anti-oxidant effects [20,53]. 

More specifically, a single xantophyll named *β*-carotene recently showed potential to play a role in dietary supplement-drug interactions by being able to inhibit efflux transporter proteins as well as phase 2 metabolic enzymes [20,53]. With regards to transporter proteins, it was found that *β*-carotene significantly inhibits P-gp function in a concentration-dependent manner. The mechanism of action was attributed to the capability of *β*-carotene to stimulate P-gp ATPase as well as binding to P-gp while slightly changing the protein conformation. Furthermore, it may also be important to note that *β*-carotene weakly inhibits BCRP, but to a much lesser extent than P-gp [53]. With regards to metabolism inhibition, it was shown that *β*-carotene has the ability to mildly inhibit UGT1A1, UGT1A3 and UGT 1A4, with the highest affinity being for UGT1A1 [20]. 

Both these interactions may potentially be used to improve the pharmacokinetics of drugs in patients, specifically with the concurrent use of anti-neoplastic drugs. When taking into account that *β*-carotene possesses its own anti-cancer effects, the P-gp and metabolism inhibition may serve as beneficial interactions to improve drug levels in the cancer cells while providing potential synergistic effects.

#### 3.3.3. Green Coffee Beans

Two components from green coffee bean extract namely caffeic acid and dicaffeoylquinic acid, were tested for interactions with transporter proteins. The results indicated potential modulation of pharmacokinetic parameters, which resulted in higher bioavailability of certain drugs [14,15]. 

Caffeic acid inhibited both hOAT1 and hOAT2 transporters [14]. PAH and ES were used as substrates for hOAT1 and hOAT2 and both their uptake was decreased in a dose dependent way. Complete inhibition of uptake transporters was observed at a caffeic acid concentration of 1 mM, which occurred by means of a competitive mechanism [14].

Dicaffeoylquinic acid was also tested for possible hOAT transporter modulation. Two enantiomers, 1,3- and 1,5-diacffeoylquinic acid, both showed the ability to inhibit hOAT1 and 3, with the exception that 1,5-dicaffeoylquinic acid inhibited these transporter proteins to a much lower extent. Inhibition of PAH transport was found to be 64% and 22% for the 1,3- and 1,5-enantiomer, respectively, while the transport of ES was inhibited by 41% and 35% by the enantiomers, respectively [15]. These results indicated that dicaffeoylquinic acid can influence the re-absorption of drugs that are substrates of active transporters in the kidney resulting in higher blood plasma levels for longer periods of time. The effect of 1,3- and 1,5-diacffeoylquinic acid on the hOAT4 uptake transporter was also tested and they significantly stimulated active transport of ES. This may further contribute to bioavailability enhancement due to stimulation of hOAT4-mediated drug transport [15].

### 3.4. Drug-Drug Interactions

Even though only two drugs that are taken simultaneously by a patient can already cause drug-drug interactions, poly-therapy increases the risk of clinically significant interactions to occur between drugs. One of the most common causes of adverse drug reactions is drug-drug type interactions, but these interactions may also be responsible for an increase in clinical efficacy [54].

#### 3.4.1. Ivermectin

Ivermectin is an anthelminthic drug of the avermectin class. Drug interactions of ivermectin in the presence of efflux transporter substrates were studied and indicated the ability of ivermectin to inhibit P-gp- and BCRP-related efflux. The efflux ratio of R123, a P-gp substrate, was reduced by approximately 3-fold in an excised rat intestinal tissue model by ivermectin. The same study also found that the secretory transport of danofloxacin, a BCRP substrate, was decreased almost 4-fold by ivermectin [10]. The results of this study therefore indicated that ivermectin has the ability to inhibit the efflux transporters, P-gp and BCRP. Similar efflux transporter inhibitory results were observed in human and mouse neuroblastoma cells [55].

Ivermectin inhibited the secretory transport of lopinavir and saquinavir by approximately 2.5 fold, whilst increasing the absorptive transport of lopinavir almost 3-fold. Due to the overlapping of helminthic infection and HIV in certain areas, especially sub-Saharan Africa, these drug-drug interactions between the two treatment regimens can possibly be used to the benefit of patients [56].

#### 3.4.2. Chlorambucil

Chlorambucil is an anti-neoplastic drug belonging to the DNA-alkylating class [57,58]. Drug-drug interactions with other anti-neoplastic drugs may lead to beneficial pharmacokinetic profiles, resulting in more efficient cancer treatment.

Initial studies with regards to potential pharmacokinetic interactions between chlorambucil and other drugs, demonstrated its capability to inhibit P-gp related efflux of a known substrate, 5-carboxyfluorescien, in leukemia and lymphoma cells. A concentration dependent trend was identified where 50, 100 and 200 µmol/L of chlorambucil inhibited 5-carboxyfluorescien efflux to 88.9%, 40.9% and 5.2% of the control, respectively [58]. These results were put into practice very recently when Liu et al. [11] synthesized a tumor-targeting anti-cancer agent by covalently binding delocalized lipophilic cation FF to chlorambucil (FFCLB). They found that FFCLB increased intracellular adriamycin concentrations dramatically, to an even larger extent than the known potent P-gp inhibitor, verapamil [11].

Furthermore, chlorambucil was demonstrated to inhibit the active transporter, OATP1B3, which is exclusively expressed in the liver and indirectly plays a role in the controlled release of gall bladder and pancreas secretions necessary for digestion. The inhibitory effect of chlorambucil may decrease hepatic metabolism of lipid molecules, but the effect on normal physiological processes by this inhibition is still unclear [13,57].

#### 3.4.3. Telaprevir

Telaprevir is a relatively new direct acting anti-viral, used for the treatment of hepatitis C. The literature widely reports on the capability of telaprevir to inhibit P-gp transport by competitive inhibition [13,58,59,60]. 

A comprehensive study by Kunze et al. [13] identified telaprevir as an inhibitor of organic cation transporter 2 (OCT2) and multi-drug and toxin extrusion transporter 1 (MATE1). OCT2 is a renal transporter that mediates tubular secretion via basolateral uptake, while MATE mediates tubular secretion, but via efflux from the apical side [13]. The uptake of [^3^H]N-methyl-4-phenyl pyridinium iodide (MPP^+^) via OCT2 was reduced by half in the presence of telaprevir, while the efflux transport of metformin via MATE1 was reduced 8-fold [13].

## 4. Discussion

In this paper, a collection of pharmacokinetic interactions are discussed within the broad categories of food-drug, herb-drug, dietary supplement-drug and drug-drug interactions as summarised in Table 2. 

Inhibition of efflux transporters and metabolism enzymes by co-administered compounds may lead to increased bioavailability of drugs with poor pharmacokinetic properties. Some of these interactions can potentially be coordinated by including functional excipients (i.e., compound that causes beneficial interaction) with the drug in a dosage form. This technique may even be used to lower the quantity of active drug required per administered dose due to increased bioavailability. A classic example of such an application is probenecid that is combined with certain antibiotics to prolong the circulation time of the antibiotics in the systemic circulation. However, such combinations to deliberately cause interactions must be used with caution as inhibition of metabolism and efflux may also lead to increased systemic concentrations of other chemical compounds. Furthermore, drug absorption enhancement by functional excipients has been associated with toxic effects and tissue damage, which should be avoided. 

Although some potential drug bioavailability enhancers have been identified, further research is necessary to find appropriate modulators that can be safely administered with drugs to improve their pharmacokinetic properties and ultimately also their efficacy. Due to a relative shortage of information on the clinical significance of some pharmacokinetic interactions, it is important to mention here that a need exists for future studies to provide clinical data on this topic. 

## Figures and Tables

**Table 1:** Effects of selected chemical components of *Glycyrrhiza* species on CYP450 enzyme inhibition [16,18].

Extract	Cytochrome P450 iso-enzymes ^1^								
	1A2	2A6	2B6	2C8	2C9	2C19	2D6	2E1	3A4
18*β*-Glycyrrhetinic acid	+	+	+	++	+	+	+	+	++
Glabridin	+	+	+	++	+	+	+	+	++
Glycycoumarin	++	+	++	+++	+++	+++	++	+	+
Isoliquiritigen	+	+	+	+++	+++	+	+	++	++
Licochalcone A	++	+	+	+++	+++	+++	++	++	++
Licoricidin	+	+	+++	+++	+++	+++	++	++	++

^1^ + = weak inhibition, ++ = moderate inhibition and +++ = strong inhibition.

**Table 2:** A summary of selected scientific pharmacokinetic interaction studies used in the discussions of this paper.

Type of Study	Additive Agent	Drug/Marker	Mechanism	Effect	Ref.
Ex vivo	Ivermectin	R123	P-gp	Inhibited	[10]
		Danofloxacin	BCRP		
	Selected aloe components	Cimetidine	P-gp	Inhibited	[37]
		Insulin	Tight junctions	Opened	[44]
		Didanosine	Buccal Absorption	Increased	[46]
		Ketoprofen	Transdermal delivery	Increased	[47]
In vitro	DAG	Digoxin	P-gp	Inhibited	[2]
	Emodin				
	Resveratrol	Methotrexate	P-gp, MRP2, OAT1 and OAT3	Inhibited	[5]
	Chlorambucil	Adriamycin	P-gp	Inhibited	[11]
	Telaprevir	MPP^+^	OCT2	Inhibited	[13]
		Metformin	MATE1		
	Caffeic acid	PAH and ES	OAT1 and OAT3	Inhibited	[14]
	Dicaffeoylquinic acid	PAH and ES	OAT1 and OAT3	Inhibited	[15]
	18*β*-Glycyrrhetinic acid	PAH	OAT1	Inhibited	
		ES	OAT4	Induced	
	*Selected Glycyrrhiza* species	Selected isoform-selective markers	CYP450	Inhibited	[16,18]
	Aqueous *Andropgraphis paniculata* extract	4-methylumbelliferone	UGTs	Inhibited	[19]
	*β*-Carotene	Selected isoform-selective markers	UGTs	Inhibited	[20]
		R123	P-gp	Inhibited	[53]
		Mitoxantrone	BCRP		
In vitro	Piperine	Digoxin and Cyclosporin A	P-gp	Inhibited	[28]
		Verapamil	CYP3A4		
	Tangeretin	Dasatinib	BCRP and P-gp	Inhibited	[32]
		Doxorubicin	P-gp	Inhibited	[33]
	Nobiletin	Dasatinib	BCRP and P-gp	Inhibited	[32]
	Selected aloe leaf material	Indinavir	CYP	Inhibited	[38]
		Insulin	Tight junctions	Opened	[41]
		FITC-dextran	Tight junctions	Opened	[43]
	Salvianolic acid B	Human serum albumin	Albumin	Competitive binding	[48,50]
		Bovine serum albumin			[49]
	Arabinogalactan	Bovine serum albumin	Albumin	Competitive binding	[51]
	Ivermectin	H33342 dye	P-gp and BCRP	Inhibited	[55]
		Lopinavir	P-gp posited	Inhibited	[56]
	Chlorambucil	[^3^H]-Cholecystokinin octapeptide	OAT1B3	Inhibited	[57]
		5-Carboxyfluorescein	MRP1	Inhibited	[58]
	Telaprevir	[^3^H]-Estrone 3-sulfate	P-gp	Inhibited	[59]
		Calcein assay			[60]
In vivo	DAG	Ketoconazole	CYP3A4/5	Inhibited	[2]
	Resveratrol	Methotrexate	P-gp,OAT1 and OAT3	Inhibited	[5]
		Chlorzoxazone	CYP2E1	Inhibited	[34]
		Diclofenac	CYP2C9	Inhibited	[35]
	Piperine	Emodin	UGT	Inhibited	[29]
		Docetaxel	P-gp and CYP3A4 posited	Inhibited	[30]
	Selected aloe leaf material	Indinavir	CYP	Inhibited	[38]
		Vitamins C and E	Intestinal absorption	Increased	[45]
